# From the Operating Room to the Dental Chair: Collaborative Prosthodontics in Craniofacial Rehabilitation

**DOI:** 10.7759/cureus.89095

**Published:** 2025-07-30

**Authors:** Shubham K Srivastava, Chinmoy Sikdar, Akshim Rana, Shitij Srivastava, Abhinav Shekhar

**Affiliations:** 1 Department of Prosthodontics, Sardar Patel Post Graduate Institute of Dental and Medical Sciences, Lucknow, IND

**Keywords:** dentistry, maxillofacial prosthodontics, oncology, prosthodontic rehabilitation, trauma

## Abstract

Craniofacial rehabilitation demands a multidisciplinary approach where prosthodontists play a pivotal yet often underutilized role in the continuum of care. Traditionally, prosthetic rehabilitation is considered a postsurgical step, leading to delays in functional restoration and compromised outcomes. This editorial emphasizes the need to redefine prosthodontics as an integral component of craniofacial surgical planning, from the operating room to the dental chair. By fostering collaboration between surgeons, prosthodontists, and digital design teams at the preoperative stage, patient-specific obturators (prosthetic devices designed to close palatal or facial defects), stents (used to maintain surgical openings or support tissue healing), and implant-supported frameworks can be planned, thereby improving surgical precision and postoperative function. Advances in 3D printing, virtual surgical planning, and CAD/CAM technology further strengthen the feasibility of this collaborative workflow. Additionally, the integration of artificial intelligence in anatomical segmentation, prosthesis design, and predictive surgical planning is a rapidly developing domain, promising greater accuracy and personalization. Despite these technological advances, barriers such as logistical challenges, reimbursement limitations, and a lack of structured interdepartmental pathways continue to hinder early prosthodontic involvement. Addressing these systemic issues through institutional protocols, interdisciplinary training modules, and streamlined communication pathways can enhance overall care delivery. Highlighting cases from oncology, trauma, and congenital deformity management, this piece calls for institutional mandates that support early prosthodontic integration to optimize both esthetic and functional outcomes in craniofacial rehabilitation, while also embracing emerging technologies that shape the future of this field.

## Editorial

Craniofacial rehabilitation, whether following oncologic surgery, traumatic injury, or congenital defect correction, remains one of the most complex and multidisciplinary domains in modern healthcare. It requires coordinated involvement from maxillofacial surgeons, plastic and reconstructive teams, speech and swallowing specialists, and prosthodontists. Among these, prosthodontics is frequently relegated to the end of the treatment timeline, engaged only after surgical intervention is complete. This siloed approach not only prolongs patient recovery but also limits the potential for optimal functional and esthetic restoration. In this editorial, we highlight the importance of involving prosthodontics in the preoperative and intraoperative phases of craniofacial rehabilitation, emphasizing a multidisciplinary approach to enhance outcomes and streamline the care continuum [1-3].

Prosthodontic rehabilitation is often viewed as a restorative solution delivered only after primary healing has occurred. However, the consequences of this deferred involvement are significant. Without early input from prosthodontists, surgical teams may be forced to make anatomical decisions without consideration for future prosthetic integration. For example, flap reconstructions, bony resections, or zygomatic placements conducted without prosthodontic guidance can hinder the alignment, stability, or esthetics of the final prosthesis. Conversely, when prosthodontists are involved early in surgical planning meetings and preoperative imaging, they can contribute to critical decisions regarding occlusal relationships, inter-arch space, retention mechanics, and prosthesis-friendly surgical guides. This collaborative model has demonstrated increased prosthesis predictability and improved patient satisfaction across various institutions globally [1-2].

In oncologic cases, especially those involving the maxilla and mandible, the role of surgical obturators, interim prostheses, and implant planning is paramount. An obturator - an intraoral prosthesis used to close palatal or maxillary defects - designed before surgery allows for immediate postoperative rehabilitation, preserving speech, swallowing, and facial contour [1]. Similarly, zygomatic and pterygoid implants may be placed during ablative surgery, reducing the total treatment timeline and improving osseointegration due to reduced micromotion. Without early prosthodontic involvement, these opportunities are missed, and secondary surgical interventions are often required, subjecting patients to additional cost, morbidity, and psychological distress [3-4].

Technological advancements further reinforce the feasibility and necessity of early collaboration. Three-dimensional imaging, virtual surgical planning (VSP), and CAD/CAM-fabricated prostheses enable detailed, patient-specific modeling of both surgical and restorative components. Additionally, artificial intelligence is increasingly being incorporated into these digital workflows, optimizing implant placement, prosthesis design, and predictive modeling of surgical outcomes. Virtual planning sessions can now involve prosthodontists, surgeons, and bioengineers working together in real time to design resection margins, reconstructive plates, and custom obturators or implants in a single streamlined workflow. These tools are not merely adjuncts but represent a paradigm shift in how craniofacial rehabilitation is conceptualized and executed [4].

The relevance of this integrated model extends beyond oncology. In trauma cases in which midface or mandibular fractures alter occlusion and dentition, early prosthodontic assessment can assist in fabricating surgical splints or guidance prostheses. Similarly, in congenital craniofacial anomalies, such as cleft lip and palate or hemifacial microsomia, the timing and design of orthopedic appliances and interim prostheses must coincide with surgical milestones. Coordinated care between the operating room and the dental chair ensures timely intervention, minimizes complications, and promotes normal development in pediatric patients [1,3].

Importantly, prosthodontic rehabilitation also encompasses the fabrication of extraoral prostheses, such as orbital, auricular, and nasal replacements, which are crucial for patients who have undergone extensive facial resections. These prostheses serve not only to restore physical appearance but also to address the profound psychological and social impacts of facial disfigurement. Early involvement of prosthodontists in the planning of these cases enables more accurate tissue modeling and better integration with surgical outcomes, ultimately improving patient self-esteem, confidence, and reintegration into daily life [2,5].

These benefits are well-documented in the literature. For instance, Sindi et al. reported significant improvements in both quality of life and psychological well-being among maxillectomy patients who underwent early obturator rehabilitation [6]. Similarly, studies involving oral cancer survivors have shown statistically significant improvements across various domains of the Oral Health Impact Profile (OHIP) and in the overall quality of life following prosthodontic intervention, as seen in research by Matapathi et al [7]. A clinical case presented by Salomão et al. further highlights the functional and esthetic advantages of early, coordinated multidisciplinary planning in craniofacial rehabilitation [8]. Plana et al. also emphasized the critical role of prosthodontics and maxillofacial prosthetics in the success of facial transplantation, reinforcing the importance of integrating dental specialists from the early stages of complex reconstructive treatment planning [9]. Additionally, Ignatius et al. demonstrated the efficacy of teleconsultation in dental prosthetics and oral rehabilitation via videoconferencing, which facilitates interdisciplinary collaboration between prosthodontists and maxillofacial surgeons - even across institutions - thus improving access to timely, expert care [10].

Despite the clear benefits, systemic barriers persist. In many hospital systems, prosthodontists are not part of the routine craniofacial or head and neck tumor board. Prosthodontic departments may lack access to surgical scheduling or intraoperative suites. Furthermore, reimbursement models often separate surgical and prosthetic phases, disincentivizing joint planning. These logistical challenges must be addressed through institutional policy changes, interdisciplinary training modules, and awareness campaigns that elevate the visibility of prosthodontic contributions. The establishment of standardized clinical pathways and multidisciplinary planning protocols would ensure continuity of care and reduce variability in outcomes [3,5]. Potential solutions include the creation of shared billing models, scheduling access across departments, and joint surgical-prosthodontic planning protocols, which are currently under development in select institutions.

Prosthodontic education also requires a shift in focus. While postgraduate training in maxillofacial prosthodontics exists, few programs offer hands-on surgical planning experience or digital design integration. Training modules must be updated to include collaborative virtual planning, soft tissue modeling, implant navigation systems, and exposure to the interdisciplinary workflow typical of craniofacial teams. Additionally, curricula should emphasize communication, systems-based practice, and leadership within interdisciplinary care models to better prepare prosthodontists for collaborative roles. By doing so, we ensure that the next generation of prosthodontists is not merely skilled in fabrication but is integral to patient-centered planning [2,4].

This editorial calls for the formal integration of prosthodontists into craniofacial surgical teams from the outset of patient care. By participating in preoperative planning, guiding intraoperative decisions, and facilitating immediate or early prosthesis placement, prosthodontists can dramatically influence the trajectory of rehabilitation. Institutions must develop joint care protocols that position prosthodontics as a parallel, not sequential, discipline in surgical rehabilitation. Only by aligning the expertise of surgical and restorative professionals can we deliver care that is not only functional and esthetic but also truly transformative [1-5].

In conclusion, the pathway from the operating room to the dental chair should no longer be linear and delayed but simultaneous and collaborative. Craniofacial rehabilitation is too complex to be fragmented; it demands a cohesive, protocol-driven partnership between surgeons and prosthodontists. Integrating prosthodontics from the very beginning of surgical planning is increasingly recognized as essential for achieving optimal patient outcomes [1].
